# Susceptibility to biofilm formation on 3D-printed titanium fixation plates used in the mandible: a preliminary study

**DOI:** 10.1080/20002297.2020.1838164

**Published:** 2020-10-29

**Authors:** Lukasz Palka, Justyna Mazurek-Popczyk, Katarzyna Arkusz, Katarzyna Baldy-Chudzik

**Affiliations:** a Private Dental Practice, Zary, Poland; b ScienceBioTech, Wrocław, Poland; c Department of Microbiology and Molecular Biology, Institute of Health Sciences, Collegium Medicum, University of Zielona Góra, Zielona Góra, Poland; d Department of Biomedical Engineering, Institute of Materials and Biomedical Engineering, Faculty of Mechanical Engineering, University of Zielona Góra, Zielona Góra, Poland

**Keywords:** Fixation plate, 3D printing, biofilm formation, bacterial contamination, surface topography

## Abstract

**Background:** In the oral and maxillofacial surgery, fixation plates are commonly used for the stabilization of bone fragments. Additive manufacturing has enabled us to design and create personalized fixation devices that would ideally fit any given fracture.

**Aim:** The aim of the present preliminary study was to assess the susceptibility of 3D-printed titanium fixation plates to biofilm formation.

**Methods:** Plates were manufactured using selective laser melting (SLM) from Ti-6Al-4 V. Reference strains of *Streptococcus mutans, Staphyloccocus epidermidis, Staphylococcus aureus, Lactobacillus rhamnosus*, and *Candida albicans*, were tested to evaluate the material’s susceptibility to biofilm formation over 48 hours. Biofilm formations were quantified by a colorimetric method and colony-forming units (CFU) quantification. Scanning electron microscopy (SEM) visualized the structure of the biofilm.

**Results:** Surface analysis revealed the average roughness of 102.75 nm and irregular topography of the tested plates. They were susceptible to biofilm formation by all tested strains. The average CFUs were as follows: *S. mutans* (11.91 x 10^7^) > *S.epidermidis* (4.45 x 10^7^) > *S. aureus* (2.3 x 10^7^) > *C.albicans* (1.22 x 10^7^) > *L. rhamnosus* (0.78 x 10^7^).

**Conclusions:** The present preliminary study showed that rough surfaces of additively manufactured titanium plates are susceptible to microbial adhesion. The research should be continued in order to compare additively manufactured plates with other commercially available osteotomy plates. Therefore, we suggest caution when using this type of material.

## Introduction

In recent years, 3D printing has been fast-developing technology, allowing for the implementation of the most intricate designs. Also, in clinical medicine, additive manufacturing techniques utilizing metal powders, such as titanium, have enabled for the production of individual implants, scaffolds, or osteotomy plates used in traumatology, orthopedics and reconstructive surgery [1–4]. Methods of producing porous metals for medical devices using 3D printing, i.e. rapid prototyping (RP), require different degrees of precision, which depend on the type and form of the material, different production costs, and working in different environments (gas or vacuum). They include selective laser sintering (SLS), inject three-dimensional printing (3DP), electron beam melting (EBM), selective laser melting (SLM), and laser engineered net shaping (LENS). Among these methods, only SLS, SLM, and EBM use powder-bed fusion (PBF) technology, which allows for manufacturing of a metal structure from the powder material [5,6].

Based on to-date experience with this technology, we know that 3D-printed rationally designed devices meet physical, mechanical, and biological standards. It has been observed that these features do not result not so much from the production technology but the biological properties of the used materials. However, long-term biological requirements, especially biocompatibility and resistance to bacterial contamination, may be problematic. Titanium is considered to be the most biocompatible material thanks to its high corrosion resistance [7–9]. On the other hand, it has been reported that its particles may diffuse to the surrounding tissues causing allergic reactions, cytotoxicity, or pro-inflammatory responses [7–14]. Therefore, studies involving titanium alloys must be conducted with high accuracy.

Based on *in vitro* and *in vivo* studies, it has been established that roughness and pore size of biomaterial scaffolds play a critical role in bone formation: adequately selected parameters result in greater bone ingrowth [15–17]. Pore sizes of 300 µm to 800 µm and roughness from 1 µm to 2 µm (Sa value) are recommended due to the formation of capillaries and enhanced bone formation [15–17]. However, these features have some disadvantages: high porosity results in diminished mechanical properties like an elastic module, and highly roughened spatial surfaces can enhance bacterial adhesion [15–17]. Bacterial contamination and bacterial biofilm may act in two ways depending on their composition. The first is causing inflammation of the peri-implant tissue (implant-associated infection – IAI) that may develop into a generalized infection. In regards to complications with fixation plates and other titanium devices like orthopedic implants, *S. aureus* and *S. epidermidis* are the key players. It has been reported that *Staphylococcus aureus* and *Staphylococcus epidermidis* are significantly involved in infections related to medical titanium implants as they have the ability to attach to most types of titanium surfaces [18–26]. The second aspect of bacterial contamination is its influence on the rate of degradation of the titanium surfaces of implants by destroying their TiO_2_ anti-corrosive coating through the secretion of substances such as lactic acid or hydrogen peroxide [7,27,28]. It is, therefore, vital for the long-term sustainability of additively manufactured titanium devices to protect the surface against bacterial contamination. Among other species present in the oral cavity, *Streptococcus mutans* and *Lactobacillus rhamnosus* are especially important due to their ability to release lactic acid, decrease the pH, and survive in acidic environments, thereby becoming dominant corrosive microorganisms [29–36]. In addition to bacteria, it is worth mentioning *Candida albicans*, because of its symbiotic relations with *S. mutans* under biofilm formation. This leads to increased glucan production, enhanced adherence, and increased biofilm complexity amplifying their harmful effect and hinders the effectiveness of antimicrobial substances [29,33–36]. Therefore, these five species of microorganisms have been chosen in this study of biofilm formation on the 3D-printed raw fixation plates.

We have to understand how different types of bacteria react on such surfaces depending on the type of implant used and its location in the body. There have been numerous attempts to use printed implants to treat mandibular fractures [37–43] along with some biocompatibility tests [43–45]. Still, there has been little research investigating the susceptibility of such materials to biofilm formation, and they are limited to a few bacterial species like *S. aureus* [46,47] and *Pseudomonas aeruginosa* [48].

This preliminary study aimed to assess the attachment and biofilm growth model of the chosen bacteria, and fungus on 3D-printed rough titanium fixation plates using microbiological and scanning electron microscopic (SEM) methods. It is a preliminary study that would lead to the development of a suitable 3D-printed material, meeting high requirements for biomaterials which could be used in personalized mandible surgery.

## Material and methods

### Materials model preparation and general characteristics

Fracture fixation plates were designed and additively manufactured using a selective laser melting (SLM) 3D printer from Ti-6Al-4 V. The surface area of the plates was tested with 3D ATOS III Triple Scanner (GOM) and it amounted to 183.55 ± 5.5 mm.

A FlexAFM atomic force microscope with an Easyscan 2 controller (Nanosurf, Switzerland) in noncontact mode was used to investigate the morphology on smaller length scales. For AFM measurements, SICONA-10 cantrivalers (AppNano, USA) were used. The measurements were corrected for typical artifacts like line displacement of the plane inclination and were illustrated by using the Gwyddion software package. The study was carried out with three different samples produced following the same procedure with five randomly selected locations on the surface of the fixation plates for the scan area of 5 µm × 5 µm, with a resolution of 512 by 512 points. The amplitude and slope parameters were calculated automatically using the EasyScan 2.0 software with the following formulas:

**Figure:**
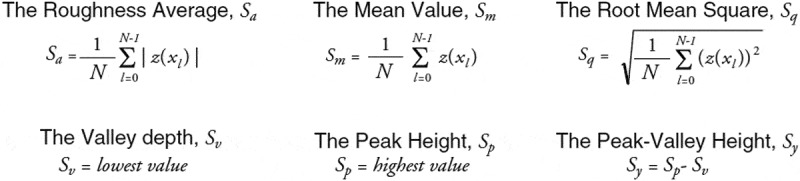

### Microbiological tests

#### Bacterial strains

Susceptibility of the material to biofilm formation was tested by using reference strains capable of forming biofilm: *S. mutans* ATCC25175, *S. epidermidis* ATCC35984; *S. aureus* ATCC29213, *L. rhamnosus* ATCC53103, and *C. albicans* ATCC10231. *S. epidermidis* and *S. aureus* were cultured in the Brain Heart Infusion medium (BHI medium; Oxoid, Thermo Scientific), *S. mutans* in BHI medium supplemented with 1% sucrose [49], *L. rhamnosus* in the de Man, Rogosa and Sharpe medium (MRS medium; Oxoid, Thermo Scientific) in an atmosphere of 5% CO_2_ with CO_2_ GenCompact (Oxoid, Thermo Scientific), and *C. albicans* on the Sabouraud Dextrose Medium with 1% glucose (Oxoid, Thermo Scientific).

### Biofilm formation

All microorganisms were cultured in the appropriate solid medium (*S. epidermidis, S. aureus*, and *S. mutans* on the BHI agar, *L. rhamnosus* on the MRS agar, and *C. albicans* on the Sabouraud agar) overnight at 37°C to obtain single colonies. Next, a single colony was suspended in 10 ml of medium (*S. epidermidis, S. aureus S. mutans* in the BHI broth, *L. rhamnosus* in the MRS broth, *C. albicans* in the Sabouraud broth), and adjusted to the OD_600_ = 0.035 ± 0.005 (NanoPhotometer NP60; Implen, Germany). One ml of suspension was then transferred to the well of a flat-bottomed polystyrene plate with the sterile, autoclaved biomaterial as the substrate for the biofilm development. Cultures were incubated for 48 h at 37°C with gentle shaking at 50 rpm (ES20 Biosan, Latvia). After incubation, biomaterials were carefully transferred to new wells of a flat-bottomed polystyrene plate and gently washed twice with 1 ml of phosphate-buffered saline (PBS; Chempur, Poland) to remove non-adherent cells. All the tested strains had been previously tested for the ability to create biofilm on the surface of polystyrene plates.

#### The assessment of metabolic activity in biofilms: TCC–based assay

Biofilm formation was quantified by a modified colorimetric method using 2,3,5-triphenyl tetrazolium chloride (TTC) as an indicator of viable bacteria [50–52]. The TCC test relies on the reduction of the colorless and water-soluble TCC to an insoluble red compound – formazon. This reduction occurs as a consequence of hydrogen ions donated to the TCC upon dehydrogenase activity in metabolically active cells. In the procedure, after biofilm formation, the biomaterial was carefully washed as described earlier and transferred to new wells of polystyrene plates filled with 1 ml of fresh medium and 20 μl of 1% TTC (Oxoid, Thermo Scientific) was added. The sample was incubated for 1.5 h at 37°C with shaking at 120 rpm. Then, the TCC solution was removed, and the sample was rinsed twice in PBS and transferred to new wells filled with 1 ml of 96% methanol (POCH). Finally, the samples were left on a shaker at 120 rpm for 15 min at room temperature. After the dissolution of the formazan crystals, the absorbance was measured on a spectrophotometer BioMate 3 (ThermoElectroCorporation) at the wavelength of 470 nm. Negative controls were biomaterial samples incubated in sterile media according to individual bacterial species. To assess biofilm formation for each tested strain and negative control, the arithmetic mean of absorbance and standard deviation were used.

#### CFU – based assay

A number of biofilm-forming bacteria were quantified through the extraction of cells from the biomaterial by mild detergent-saponin [50]. Biomaterial samples, coated with a 48-h biofilm after rinsing, were transferred to 1 ml of a 0.5% saponin solution (Pol-Aura, Poland) and incubated at 37°C for 30 min. Then, the samples were shaken at 120 rpm for 1 h to detach the bacterial cells mechanically from the biomaterial surface mechanically. The resulting bacterial suspensions were serially ten-fold diluted. One hundred µl of each of the dilutions was inoculated onto the appropriate agar medium and incubated at 37°C for 24 hours. Obtained colonies were counted, and the number of colony-forming units (CFU/ml) was calculated. Experiments were performed in triplicate to calculate the average value.

#### SEM analysis

The microscopic analysis SEM/EDS of the brand new and microbiologically tested models of raw plates was performed using the scanning electron field-emission microscope JEOL JSM 7600 F equipped with an X-ray analyzer INCA OXFORD. The microscopic observation of the biological layer required the use of an additional sample preparation procedure [53] which included immersing samples in a 3% solution of glutaraldehyde (25% in H_2_O, Grade I) in a phosphate buffer (0.05 mmol/l, pH 7.2 at 25°C, Sigma Aldrich, Ireland) and rinsing three times for 15 min in a phosphate buffer solution (0.01 M PBS; pH 7.4) at room temperature. The solution of glutaraldehyde in phosphate buffer was used for the fixation of the biological layer on raw plates. Glutaraldehyde influences the production of crosslinks between different chemical groups of the specimen and the creation of methylene bridges. As a result, the structure of this product was more bounded and stiffened [54]. Then, the samples were dehydrated in acetone solutions with increasing concentrations (% v/v 10, 20, 30, 40, 50, 60, 70, 80, 90) for 10 min. The final dewatering was carried out in 100% acetone, twice for 30 min. These operations occurred at room temperature. Further, samples were dried at critical CO_2_ points using the critical point dryer CPD E3000 (Quorum Technologies Ltd). Each chemical was purchased from Sigma Aldrich, Ireland.

### Statistical analyses

A graphic image comparing the activity of the tested strains in biofilm formation was obtained using the GraphPad PRISM version 5.01. The comparison of the differences in the results obtained with the use of the quantitative and qualitative methods was assessed applying the Spearman correlation coefficient.

## Results

Printing parameters of the fracture fixation plates are presented in Table 1. The material composition of the microstructural analysis is shown in Table 2.

**Figure 1.**
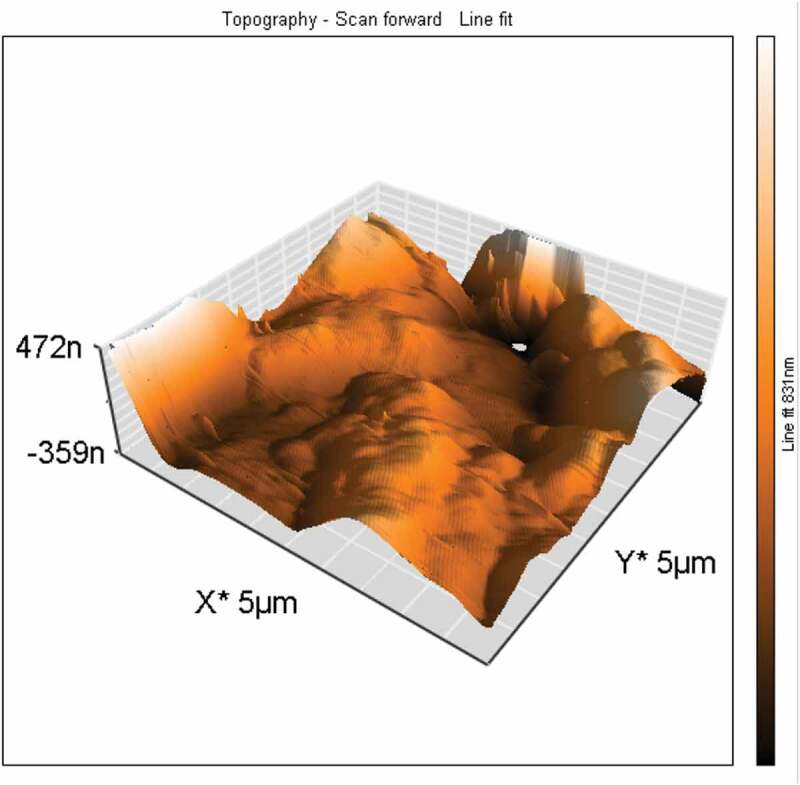
The topography of the surface of the fixation plates

**Table 1.** The collective representation of the results of chemical composition microstructure analysis

Ti [%]				Al. [%]				V [%]		
min	max	average		min	max	average		min	max	average
87.30	90.28	89.02		6.83	7.81	7.29		2.63	4.90	3.69

Ti- titanium; A- aluminum; V–vanadium.

**Table 2.** General printing parameters

Parameter	Norm	Range (after heat treatment)
Standard accuracy [mm]	-	± 0.2% (± 0.2)
Layer thickness [mm]	-	0.03 ÷ 0.6 (used 0.034)
Maximum dimension of the part [mm]	-	245 x 245 × 270 mm
Ultimate tensile strength	DIN EN ISO 6892–1:2009	1029 ± 80 MPa
Ultimate elongation [%]		14 ± 0.4%
Young’s modulus [GPa]	DIN EN ISO 6892–1:2009	104 ÷ 124
Impact strength [J]	-	7 ÷ 15
Hardness [HV5]	DIN EN ISO 6507–1	320 ± 15
Relative density [%]	-	> 99.5
Density [g/cm^3^]	-	4.41
Max. operating temperature [°C]	-	350

The surface topography analysis by using the atomic force microscope revealed an average roughness of 102.75 ± 6.03 nm (Table 3) of the printed plates and irregular topography (Figure 1).

**Figure 2.**
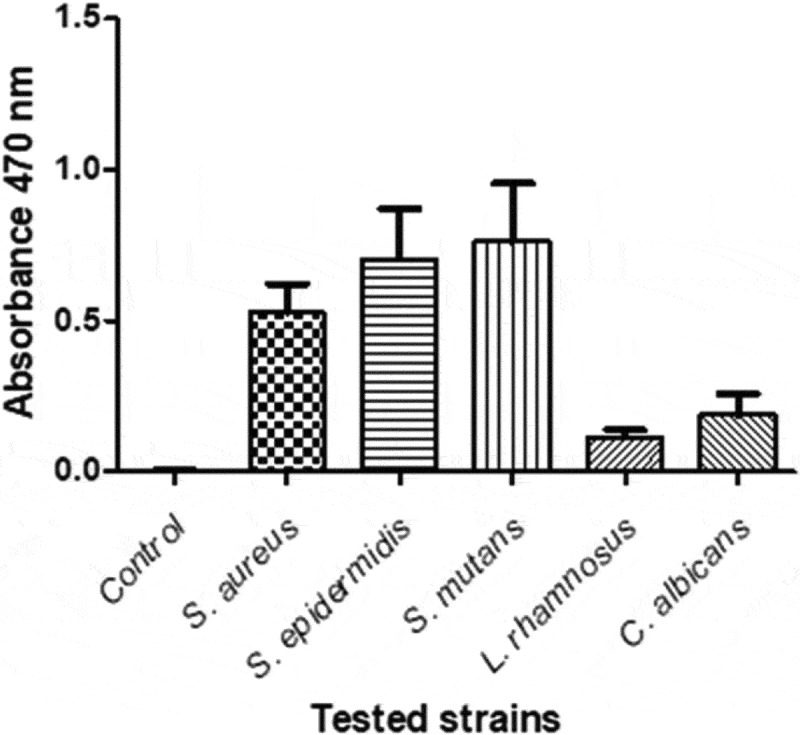
Mean values and standard deviation of the assessment of metabolic activity in biofilms using TTC assay. This test demonstrated that after 48 h of incubation, all tested microorganisms present on the surface of the fixation plates showed metabolic activity in biofilms, but in a differentiated manner (Figure 2)

**Table 3.** The mean values of surface roughness parameters, measured for three samples at five different places

Area [pm^2^]	Sa [nm]	Sq [nm]	Sy [nm]	Sp [nm]	Sv [nm]	Sm [pm]
25.20	102.75 ± 6.03	134.92 ± 6.80	1187.40 ± 88.16	602.35 ± 29.08	−585.06 ± 31.04	−0.0031 ± 0.0001

Sa – the difference in height of each point compared to the arithmetical mean of the surface; Sq – the root mean square value of the ordinate values within the defined area. It is equivalent to the standard deviation of heights; Sp – the height of the highest peak within the defined area; Sv – the absolute value of the height of the largest pit (valley) within the defined area; Sm – the mean spacing between peaks.

*S. mutans* and S. *epidermidis* strains showed a very strong metabolic activity in biofilms compared to the other strains tested. *S. aureus* strain showed lower activity in relation to *S. mutans* and *S. epidermidis. C. albicans* and *L. rhamnosus* strains showed clearly lower activity in the biofilm compared to the other tested strains.

A saponin test was used to assess the number of biofilm-forming cells on the surface of a raw 3D-printed plate. Saponin activity, combined with intense shaking led to the complete removal of biofilm-forming cells from the surfaces of the plates. This method allowed us to assess the average cell number of individual strains of microorganisms forming biofilm on the surface of the tested plates (Table 4). Disclosed variations in the cell number of each strains of microorganisms may be presented as follows: the average CFU of strains from *S. mutans* (11.91 x 10^7^) > *S. epidermidis* (4.45 x 10^7^) > *S. aureus* (2.3 x 10^7^) > *C.albicans* (1.22 x 10^7^) > *L. rhamnosus* (0.78 x 10^7^).

**Table 4.** Average values of the number of cells recovered from the biofilm formed on the surface of the fixation plate model

Strains from	*S. aureus*	*S. epidermidis*	*S. mutans*	*L. rhamnosus*	*C. albicans*
Mean number of microbial cells CFU/plate (CFU/sample)	2.3 x 10^7^	4.45 x 10^7^	11.9 x 10^7^	0.78 x 10^7^	1.32 x 10^7^

Comparison of the results of two methods showed the highest correlation coefficient for *L. rhamnosus* (0.7897). The results of biofilm formation evaluation for *C. albicans* (0.7599), *S. epidermidis* (0.7009) and *S. aureus* (0.6825) were characterized by high correlation coefficients. The lowest Spearman’s correlation coefficient (0.5042) was obtained for the results of biofilm assessment with *S. mutants*.

**Figure 3.**
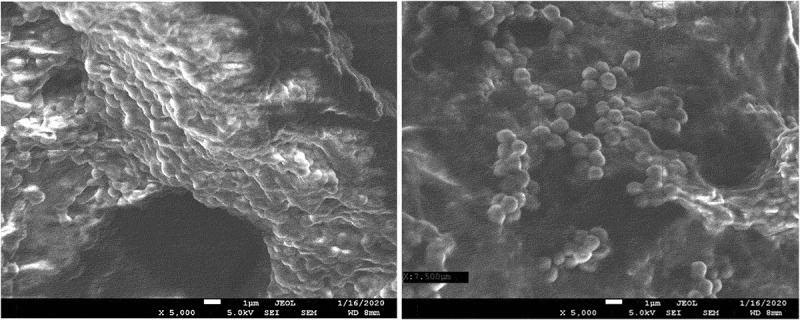
(**a**), (**b**). Scanning electron microscopic image showing massive colonization by *S. mutans.*

**Figure 4.**
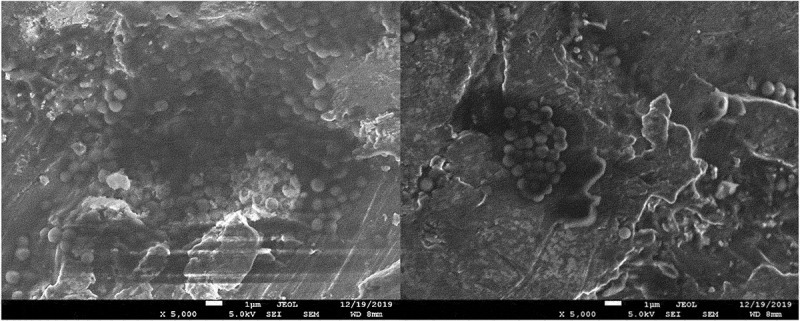
(**a**), (**b**). Scanning electron microscopic image showing biofilm of *S. epidermidis.*

**Figure 5.**
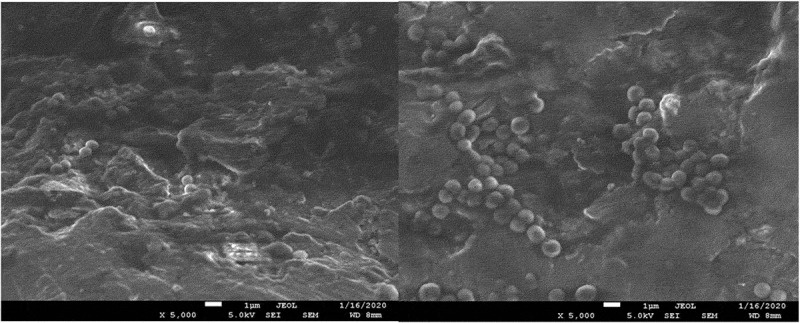
(**a**), (**b**). Scanning electron microscopic image showing biofilm of *S. aureus.*

**Figure 6.**
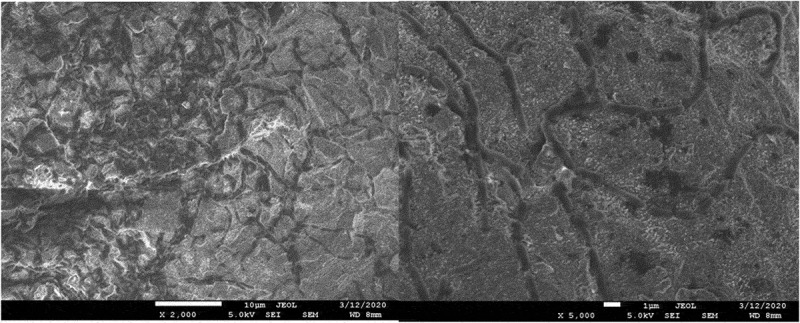
(**a**), (**b**). Scanning electron microscopic image showing biofilm of *L. rhamnosus.*

**Figure 7.**
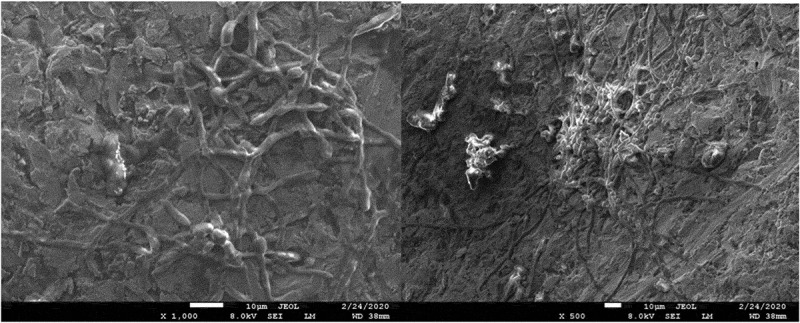
(**a**), (**b**). Scanning electron microscopic image showing biofilm of *C. albicans.*

The results of the microbiological tests were confirmed by visualization of bacterial cells using SEM microscopy. The analysis showed both the surface shape of the 3D-printed plates and the specific manner of growth for each of the tested species after 48 hours of culture. *S. mutans* formed dense biofilms covering most of the titanium plate surface (Figure 3). *S. epidermidis* also created conglomerates on the plate surface, mostly covering the depressions (Figure 4). Smaller conglomerates and sparse cells were revealed in the biofilm of *S. aureus* (Figure 5). SEM images show *L. rhamnosus* cells forming a characteristic biofilm network. Newly formed cells lengthened the network by locating in the wells/depressions of the plate (Figure 6). Extensive and highly structured biofilm with the filamentation was observed in the case of *C. albicans* growth (Figure 7).

## Discussion

In oral and maxillofacial surgery, fixation plates are commonly used for the stabilization of bone fragments in trauma management. Currently, implants made of titanium or its alloys are most often used in routine treatment. 3D printing has enabled the preparation of fracture fixation material customized specifically for each patient. Medical applications for 3D printing have evolved considerably and are expected to revolutionize part of the health care in the nearest future [37]. Regardless of the great advancement in biomaterial technology, introduction of such biomaterials into a living organism, like any other implant, may result in implant-related infections [55]. Biomaterial contamination during surgery may lead to the initial adhesion of the microorganisms. Unfortunately, the metallic surface of the implant absorbs proteins such as fibronectin, which facilitates microbial adhesion and biofilm formation. Biofilm formation is a dynamic process that is influenced by a number of factors, e.g. the type of surface to which microbes adhere or the specific properties of these microorganisms [56]. Commonly Identified microorganisms causing infections associated with fracture-fixation devices are mainly *S. aureus*, coagulase-negative staphylococci such as *S. epidermidis*, Gram-negative bacilli, anaerobes, enterococci and streptococci [57]. According to Campoccia et al., two species, *S. aureus* and *S. epidermidis* account for around two-thirds of implant infections [58], and approximately 20% of all orthopedic device-related infections, increasing up to 50% in late-developing infections [59]. Infections that occur in the mandible, also have a direct relationship with the biofilm created by microorganisms of the oral cavity, like *S. mutans* and lactobacilli.

Due to the limited data on the susceptibility of the 3D-printed plates to the adhesion and biofilm formation, our model study aimed to assess the adhesion and growth of *S. aureus, S. epidermidis, S. mutans, L. rhamnosus* and *C. albicans* biofilm on a 3D-printed Ti-6Al-4 V raw plate. In the present study, a 48-h biofilm formation was assessed by microbiological methods. The results have shown that the ability to attach and develop biofilms is strain-dependent. The strain of *S. mutans* showed stronger ability than other strains (namely those of *S. epidermidis, S. aureus, C. albicans*, and *L. rhamnosus*) to adhere to the rough surface of the titanium plate used in the model.

Factors which influence bacterial adherence and biofilm formation on biomaterials include their chemical structure and surface roughness. The irregular rough surface of biomaterials, especially their micro and macro-structures improve osseointegration which is a desirable process in implantology. Surface roughness and topography have a significant correlation with bone regeneration and mechanical retention in the human maxilla and mandible [60] but they also facilitate initial microbial adhesion and the formation of biofilms [61].

Obtained results are in accordance with previously reported findings on the area roughness of parts produced using the metal-based 3-D printing process [62,63], and have vertical faces with a typical roughness 50% greater than the horizontal faces [64]. The average roughness (Sa) and the root mean square (Sq) parameters seem to be significantly lower than described in the literature [65], due to the lower pore area (5 x 5 um) which was chosen to give a more detailed description of the interactions between the surface and the bacteria. The presence of any surface irregularities, depressions, or hollows promotes bacterial retention. *In vitro* studies have shown that the colonization of the implant surface by microorganisms begins just in the hollows that are a good niches for bacterial cells [66]. Scanning electron microscopy in our studies also revealed that bacterial biofilms tended to form in crevices.

Mello et al. conducted biofilm studies on the Ti-6Al-4 V alloy produced by means of powder metallurgy (pores measured 300 μm of diameter and resulted in a porosity of 40%), and obtained results comparable to ours [67]. The strongest biofilm formation was observed for *S. aureus* (5.43 × 3.38 × 10^8^ ± 0.68 × 10^8^), followed by *S. mutans* (6 × 10^7^ ± 1.58 × 10^7^) and *C. albicans* (10^6^ ± 1.16 × 10^6^); however, *S. mutans* was grown in BHI medium without the addition of sucrose, which certainly contributed to a weaker biofilm growth. In our approach adherence of *S. mutans* was tested by sucrose-dependent mechanisms, since the sucrose-independent mechanism is not relevant in the virulence of this bacterium [68]. When testing adhesion on various alloys used as biomaterials *C. albicans* creates a much weaker biofilm compared to staphylococci and streptococci [69]. This may be due to a much larger yeast cell size and more difficult initial adhesion compared to cocci, which more easily penetrate into the cavities of the porous materials. Wu et al. reported that a species of *Streptococcus* preferred concave features such as valleys, depressions and pits, all of which function to enhance the bacteria-surface area contact [70]. Additionally, Yoda et al. demonstrated that even quite a low surface roughness ranging from 7.1 to 16.5 nm Ra for Ti-6Al-4 V can influence bacterial adhesion and biofilm formation of *S. epidermidis* [71].

Lactobacilli are not often used to test biofilm formation on medical biomaterials although they constitute a part of the oral microbiota [72], are early colonizers in oral biofilms [73], and can be isolated form infected craniomaxillofacial osteosynthesis plates [74]. The present study showed the ability of *L. rhamnosus* to adhere and form biofilm on the tested fixation plates. Production of organic acids by lactic acid bacteria can additionally contribute to the corrosion of the biomaterial.

*C. albicans* is a common fungal species present in the oral cavity, which may colonize tissues but also prosthetic surfaces and implants [56,75,76]. Infection induces inflammatory reactions, and also contributes to the formation of multispecies biofilms consisting mainly of various species of streptococci [77]. The quantitative culture methods revealed the lowest values for *C. albicans* among all the tested strains, but in the scanning electron microscope the rough surfaces of fixation plates were covered with extensive and highly structured biofilm. This indicates that 3D-printed biomaterials are susceptible also to fungal adhesion.

The irregular rough surface of the fixation plates with its micro and macro-structures certainly facilitated adhesion and the formation of biofilms of all the tested microorganisms. Data regarding the relationship of the surface roughness and the ability to form biofilm are not uniform. Surfaces with Sa greater than 0.2 μm (200 nm) have been reported to facilitate biofilm growth [78]. Quirynen et al. reported that *in vivo* surface roughness below 0.2 μm did not affect bacterial adhesion [15]. Park et al. found decreased adhesion of streptococci and other species at surface roughness values of around 0.15 µm [73]. The results of *in vitro* studies conducted by Yoda et al. suggested that even a quite a low surface roughness ranging from 7.1 to 16.5 nm Ra for Ti-6Al-4 V can influence bacterial adhesion [61]. Plates tested in the present study had a lower mean Sa of 0.102 μm; however, all used strains were able to form biofilms. These discrepancies may result from the fact that clinically different prostheses or implant devices are manufactured in different manners. Thus, other parameters and factors need to be considered, like wettability, free surface energy, and surface chemical composition. Features of addictive manufactured implants depend on many factors like the size of powder particles, printing parameters, material, etc. leading to various results.

One of the attempts to reduce the susceptibility of 3D-printed material to biofilm formation can be post-processing, e.g. polishing. Xie et al. showed that discs cut from Ti6Al4V implant manufactured by using selective laser melting (SLM), had higher bacterial adhesion than polished ones [79]. It has also been suggested that metallic implants produced by laser powder-bed fusion should be polished or coated [46,47] since coating medical devices with different kinds of active agents may be equally successful [80–82]. Rodrigez-Lopez et al. reported that thin polymer multilayers composed of chitosan and hyaluronic acid which release a β-amino acid-based peptidomimetic of antimicrobial peptides (AMPs) may prevent *C. albicans* and *S. aureus* biofilm formation [81].

Nonetheless the research should be continued and involve cell culturing and *in vivo* studies, as only such complex approaches will allow us to fully understand the problem of medical devices’ susceptibility to infection and may lead to other results than *in vitro* studies. For example, Metsemakers et al. conducted *in vivo* studies in rabbits, in which they compared *S. aureus* growth on fracture fixation plates with different surface topographies. The results did not show any significant differences between titanium and steel implants with conventional or modified surface in relation to their susceptibility to infection [82]. This means that *in vivo* studies are necessary to validate *in vitro* studies as they may show different results.

## Conclusions

Titanium and its alloys are a type of biomaterials that have been increasingly used in many medical devices due to its biocompatibility, mechanical and anti-corrosive properties. Additive 3D printing technologies create an opportunity to quickly acquire and optimally match medical tools to the patient’s needs. In these studies, we have shown that the raw, rough surface of the fixation plate obtained by 3D technology promotes adhesion and biofilm formation by various microbiota. It has been shown that the ability to adhere and form biofilm can vary between strains of *S. aureus* and *S. epidermidis*, which are potential surgical contaminating pathogens, and of *S. mutans, L. rhamnosus*, and *C. albicans*, which are parts of the oral microbiota. These preliminary studies underscore the importance of the surface properties of raw 3D-printed plates, which can promote bacterial adhesion and biofilm formation. Further studies comparing a raw, rough surface with a polished or modified surface may reveal more relationships between the surface character of the 3D-printed plate and the ability of bacteria to form biofilm.
